# Decision-Making and Downstream Outcomes of the Gabapentinoid-Diuretic Prescribing Cascade

**DOI:** 10.1001/jamanetworkopen.2025.45274

**Published:** 2025-12-02

**Authors:** Matthew E. Growdon, Natalie Tjota, Rachel Campbell, Phyllis Gayda, Bocheng Jing, W. James Deardorff, Lisa M. McCarthy, Kenneth S. Boockvar, Michael A. Steinman

**Affiliations:** 1 Division of Geriatrics, University of California, San Francisco; 2 San Francisco Veterans Affairs Medical Center, San Francisco, California; 3 Leslie Dan Faculty of Pharmacy, University of Toronto, Toronto, Ontario, Canada; 4 Institute for Better Health, Trillium Health Partners, Mississauga, Ontario, Canada; 5 Division of Gerontology, Geriatrics, and Palliative Care, University of Alabama at Birmingham; 6 Geriatrics Research, Education, and Clinical Center, Birmingham VA Health Care System, Birmingham, Alabama

## Abstract

**Importance:**

Prescribing cascades are an underrecognized driver of polypharmacy among older adults (aged ≥65 years). The clinical decision-making processes underlying cascades and their downstream consequences are poorly understood.

**Objective:**

To explore clinical reasoning leading to prescribing cascades and downstream outcomes (eg, falls, electrolyte abnormalities) via the exemplar gabapentinoid (gabapentin and pregabalin)–loop diuretic (LD) cascade.

**Design, Setting, and Participants:**

This cohort study randomly selected medical records from a cohort of US veterans aged 66 years or older between January 1, 2013, and August 31, 2019, who potentially experienced the gabapentinoid-LD prescribing cascade. The medical record review and data analysis were performed between October 24, 2023, and July 22, 2025.

**Exposures:**

Initiation of gabapentinoid and LD.

**Main Outcomes and Measures:**

Abstractors evaluated clinical documentation in the 30 days prior to and 60 days after LD initiation to evaluate decision-making processes and potential downstream outcomes of the gabapentinoid-LD cascade. Secondary analyses examined whether a dementia diagnosis was associated with clinician decision-making and patient outcomes.

**Results:**

The analytic cohort comprised 120 patients (mean [SD] age, 73.9 [7.1] years; 116 male [96.7%]; 106 [88.3%] taking ≥5 long-term medications). Documentation of a differential diagnosis for edema was noted in 73 patients (60.8%), most commonly referencing congestive heart failure (n = 47 [39.2%]) and/or venous stasis (n = 16 [13.3%]). Gabapentinoids were rarely noted in the differential (n = 4 [3.3%]). The majority of clinicians documented the indication for LD (n = 116 [96.7%]), most commonly for lower-extremity edema (n = 104 [86.7%]), congestive heart failure (n = 16 [13.3%]), and/or dyspnea (n = 15 [12.5%]). In the 60 days following LD initiation, 28 patients (23.3%) experienced 37 events potentially attributable to LD initiation. The most common downstream events were worsening kidney function (n = 9 [7.5%]), orthostasis (n = 7 [5.8%]), electrolyte abnormalities (n = 6 [5.0%]), and falls (n = 5 [4.2%]). Six patients (5.0%) were evaluated in the emergency department and/or hospital for potential downstream events. Documentation of differential diagnoses, indications, actions taken regarding gabapentinoids, and downstream events generally did not vary between patients with and without dementia.

**Conclusions and Relevance:**

This cohort study found that among older veterans who received LD following gabapentinoid initiation and experienced a potential gabapentinoid-LD prescribing cascade, clinicians almost never explicitly considered gabapentinoid adverse drug effects in their treatment of edema. These findings suggest that potential downstream harms of this overlooked prescribing cascade are common, underscoring the importance of addressing prescribing cascades in clinical practice.

## Key Points

**Question:**

What are the key clinician decision-making steps in the gabapentinoid (gabapentin and pregabalin)–edema–loop diuretic (LD) prescribing cascade and its downstream outcomes?

**Findings:**

In this cohort study of 120 veterans aged 66 years or older who potentially experienced the gabapentinoid-LD prescribing cascade, clinicians almost never documented consideration of gabapentinoid adverse drug effects in their management of edema. Potential downstream harms, including worsening kidney function, electrolyte abnormalities, and falls (some precipitating emergency department visits and/or hospitalizations) were common among patients initiating both a gabapentinoid and an LD.

**Meaning:**

These findings suggest that prescribing cascades may propagate unintentionally and potentiate important drug-related harms among older adults.

## Teaser

This cohort study explores the clinical decision-making process influencing the gabapentinoid–edema–loop diuretic prescribing cascade and its downstream outcomes among older US veterans.

## Introduction

Prescribing cascades occur when a medication causes adverse effects that are treated with a second medication and are an increasingly recognized driver of polypharmacy among older adults (aged ≥65 years).^1,2^ Existing research on prescribing cascades has largely focused on the identification of various prescribing cascades using administrative data and, to a lesser extent, delineating subsequent health impacts.^1,3,4,5,6^ Since first conceptualized,^7^ the concept of prescribing cascades has undergone refinement. For example, prescribing cascades may be characterized based on their intentionality (eg, whether the adverse effect of the first drug is recognized) and appropriateness (eg, balance of risk and benefits associated with the prescribing cascade).^8^

Despite growing recognition of the importance and nuances of prescribing cascades, little is known about how and why prescribing cascades occur and the full extent of their downstream outcomes. Initial qualitative work has revealed that cascades are difficult to identify (by patients and clinicians), and their occurrence is associated with varying levels of awareness of medications and their adverse effects.^9,10^ Yet, to date, we lack a granular understanding of key decision-making steps that lead to prescribing cascades in clinical practice. Such information could be critical to informing nascent efforts to develop and appropriately target interventions to prevent and/or mitigate prescribing cascades.^11,12^

Using a structured medical record review of US Department of Veterans Affairs (VA) clinical data, we characterized the decision-making processes and potential downstream consequences of the gabapentinoid (gabapentin and pregabalin)–edema–loop diuretic (LD) prescribing cascade.^13,14,15^ We chose this approach for several reasons. First, the gabapentinoid-LD prescribing cascade has been confirmed in several studies.^13,14,16,17^ Peripheral edema is an established adverse effect of gabapentinoids; is experienced by 7% to 10% of patients^18,19^; and is primarily mediated by their action on presynaptic voltage-gated calcium channels, leading to altered myogenic tone and vasogenic edema.^20,21^ However, prescribers associate gabapentinoids with a relatively favorable adverse effect profile, thereby setting up a situation in which unintentional prescribing cascades may be more likely.^22^ Furthermore, LDs are associated with potential harms, including volume loss, orthostasis, electrolyte abnormalities, and falls.^23^ Second, gabapentinoid prescribing has ballooned in recent years in the US due to off-label use for pain and other conditions, increasing by 84% between 2012 and 2022.^24,25^ Gabapentinoids are among the top 10 drugs prescribed to older adults,^24^ and a better understanding their role in prescribing cascades may carry important implications for a large population of older adults.

As a secondary aim, we explored whether the presence of dementia is associated with decision-making that may lead to the gabapentinoid-LD prescribing cascade. People with dementia are particularly vulnerable to medication-related harms given their cognitive decline and high prevalence of polypharmacy.^26,27,28^ Moreover, people with dementia and their caregivers face unique challenges in reporting adverse effects to prescribers (eg, due to a lack of awareness of adverse effects and/or ability to communicate), which may translate to a different process leading to prescribing cascades and their outcomes.

## Methods

### Sample

This cohort study was approved by the institutional review boards of the San Francisco VA Health Care System and the University of California, San Francisco School of Medicine. The institutional review boards waived the need for informed consent for all study procedures because of minimal risk to patients. The study followed the Strengthening the Reporting of Observational Studies in Epidemiology (STROBE) reporting guideline for cohort studies.

We derived our sample from a previously published cohort study involving older adults receiving care from the VA who newly initiated LDs within 6 months following new initiation of gabapentinoids between January 1, 2013, and August 31, 2019.^13^ Patients were aged 66 years or older and, to exclude other common causes of edema, did not have a diagnosis of congestive heart failure (CHF), chronic kidney disease, liver disease, and/or venous insufficiency in the 1 year prior to initiation of a gabapentinoid (defined by *International Classification of Diseases, Ninth Revision* [*ICD-9*] and *International Statistical Classification of Diseases, Tenth Revision* [*ICD-10*] codes). Other inclusion and exclusion criteria have been previously described in detail.^13^ We defined new initiation of gabapentinoids and LDs with a washout period of 1 year.

From 1981 patients who initiated an LD after a gabapentinoid, we identified a random sample for structured medical record review. Given our desire to understand whether dementia plays an important role in the decision-making processes underlying potential prescribing cascades, we enriched the sample for patients with dementia (1 patient with dementia per 3 patients without dementia). In the initial phase, we excluded medical records for 3 reasons to focus on cases in which the gabapentinoid-LD prescribing cascade was plausible. First, we excluded records in which it was unclear whether the LD prescription was new for the patient. We excluded patients who were receiving drugs outside of VA pharmacies during the record review. Second, given that the gabapentinoid-LD prescribing cascade hinges on recognition of edema as an entity requiring treatment (with an LD), we excluded records in which we could not find documentation of peripheral edema in the 30 days prior to LD prescription (eg, an LD given solely for treatment of resistant hypertension). Third, we excluded records documenting that the gabapentinoid was stopped at least 30 days prior to LD, reasoning that in these cases, a gabapentinoid-LD prescribing cascade was not likely. We randomly sampled from the broader cohort until we reached a sample of 120 included records. The sample size was powered to estimate the frequencies of key medical record review elements within a 95% confidence range of 6% to 9% given an expected proportion of review elements of 5% to 50%.

### Medical Record Review Process

Trained abstractors (M.E.G., N.T., R.C., and P.G.) reviewed patient medical records using a standardized abstraction form (eAppendix in Supplement 1) and inputted data into the VA Research Electronic Data Capture database. We evaluated all clinical notes and structured data elements of the VA’s national electronic health record in the 30 days prior to and 60 days after LD initiation. Medical record review elements included documentation related to (1) a differential diagnosis for peripheral edema, (2) an indication for LD prescription, (3) actions taken toward the gabapentinoid (eg, dose reduction or discontinuation within 30 days of LD initiation), (4) history of CHF (eg, in a note or problem list but not captured in *ICD-9* and *ICD-10* definitions of CHF), (5) diagnostic studies obtained for edema, and (6) downstream events potentially associated with LD in the 60 days following its prescription. For potential downstream events (eg, falls, electrolyte abnormalities, other adverse effects listed in product labeling for LDs^23^), the abstractors conducted a causality assessment following the World Health Organization Uppsala Monitoring Centre’s system^29^ to rate the likelihood that the event could be attributed to LD use (eMethods in Supplement 1).

In the initial phase of the medical record review process, the abstractors independently reviewed the same records in batches of 10, meeting frequently to reconcile differences, promote concordance, and assess interrater reliability. In the third round of this initial phase, Fleiss κ for the presence of 5 key medical record review domains was 0.83, indicating excellent interrater reliability among the abstractors.^30^ Thereafter, each abstractor independently reviewed the remaining records. A second abstractor independently reviewed potential downstream events, with discrepancies resolved by consensus.

### Statistical Analysis

Medical record review and data analysis were performed between October 24, 2023, to July 22, 2025. We derived descriptive data (eg, age, demographics, health services use) from VA administrative data as described previously (eMethods in Supplement 1).^13^ We collected race and ethnicity (Black, Hispanic, White, and other [American Indian or Alaska Native, Asian or Pacific Islander, other, or unknown]) information based on the Research Triangle Institute definitions found in Medicare claims to provide information about included patients and the potential generalizability of the study population. We determined dementia diagnosis using *ICD-9* and *ICD-10* codes with a 3-year look-back period.^13^ Descriptive analyses evaluated the occurrence of key decision-making steps along the potential cascade for each patient. We used *t* tests, χ^2^ tests, and Fisher exact tests to compare patients with and without dementia. In an exploratory analysis, we used logistic regression to assess associations between variables, including dementia and 2 outcomes (presence of differential diagnosis and potential downstream event). Given the relatively small sample size, we parsimoniously selected variables a priori (age, comorbidity burden, and dementia diagnosis). We used SAS, version 9.4 (SAS Institute Inc) and Stata, version 18.0 (StataCorp LLC) for analyses. We considered *P* < .05 as the threshold for significance.

## Results

### Sample Characteristics

We completed medical record reviews for 200 patients (eFigure in Supplement 1). Of these, 80 patients were excluded for at least 1 reason (32 [40.0%] as it was unclear whether the LD represented a new prescription, 27 [33.8%] as there was no documentation of edema, and 26 [32.5%] as the gabapentinoid had been stopped ≥30 days before LD initiation). The analytic sample comprised 120 patients (mean [SD] age, 73.9 [7.1] years; 4 female [3.3%] and 116 male [96.7%]; 13 identified as Black [10.8%], 6 as Hispanic [5.0%], 98 as White [81.7%], and 3 as other [1.6%] race and ethnicity) (Table 1). A total of 106 patients (88.3%) were taking 5 or more long-term medications. Overall, 34 patients (28.3%) had dementia. In general, patients with dementia tended to be older (aged ≥74 years: 22 [64.7%] compared with 24 [27.9%] without dementia), to be identified as a race and ethnicity other than White (8 Black patients with dementia [23.5%] compared with 14 [16.3%] without dementia identified as Black, Hispanic, or other race and ethnicity), and to have a greater comorbidity burden (median Charlson Comorbidity Index [IQR], 4.5 [3.0-7.0] vs 3.0 [1.0-5.0] for patients with vs without dementia, respectively) (eTable 1 in Supplement 1). While the cohort was constructed to exclude individuals with *ICD-9* and *ICD-10* coding for CHF, there were 12 patients (10.0%) with medical record–documented CHF (eg, in prepopulated problem lists). Among those initiating gabapentin (n = 111 [92.5%]), the median total daily dose prior to LD initiation was 600 mg (IQR, 300-900 mg); among those initiating pregabalin (n = 9 [7.5%]), the median total daily dose was 150 mg (IQR, 100-150 mg). Among 72 patients (60.0%), the clinician who prescribed the LD was different from the clinician who prescribed the gabapentinoid.

**Table 1.** Sample Characteristics (N = 120)

Characteristic^a^	Patients, No. (%)
Age, y	
Mean (SD)	73.9 (7.1)
66-74	74 (61.7)
75-84	33 (27.5)
≥85	13 (10.8)
Sex	
Female	4 (3.3)
Male	116 (96.7)
Race and ethnicity	
Black	13 (10.8)
Hispanic	6 (5.0)
White	98 (81.7)
Other^b^	3 (2.5)
Charlson Comorbidity Index, median (IQR)	3.0 (1.0-6.0)
Dementia	34 (28.3)
Baseline medication count ≥5	106 (88.3)
Hospitalization in the past year	23 (19.2)
Clinical encounters in the past year, median (IQR), No.	25 (14-44)
Specialty clinics visited in the past year, median (IQR), No.	4 (3-6)
Different clinicians prescribing gabapentinoid and loop diuretic	72 (60.0)

^a^ Sample characteristics were derived from US Department of Veterans Affairs and Medicare administrative data.

^b^ Includes American Indian or Alaska Native, Asian or Pacific Islander, other, or unknown.

### Documentation of Key Steps Along the Potential Prescribing Cascade Following Gabapentinoid Initiation

Following gabapentinoid initiation and development of edema, documentation of a differential diagnosis for edema was noted in 73 patients (60.8%), most commonly with reference to CHF (n = 47 [39.2%]) and/or venous stasis (n = 16 [13.3%]) (Table 2; eTable 2 in Supplement 1). Nongabapentinoid drug-related adverse effects were considered in the differential for 11 patients (9.2%), most often related to calcium channel blockers (7 patients, of whom 5 stopped). Gabapentinoids were rarely noted in the differential (n = 4 [3.3%]).

**Table 2.** Key Decision-Making Steps and Potential Downstream Outcomes of the Gabapentinoid–Loop Diuretic Prescribing Cascade (N = 120)

Characteristic	Patients, No. (%)
Differential diagnosis documented for edema^a^	
Any	73 (60.8)
Congestive heart failure	47 (39.2)
Venous stasis	16 (13.3)
Nongabapentinoid adverse drug effect^b^	11 (9.2)
Deep venous thrombosis	7 (5.8)
Gabapentinoid-associated adverse effect	4 (3.3)
Other^c^	18 (15.0)
Gabapentinoid action within 30 d before diuretic initiation	
Any	8 (6.7)
Dose increased	5 (4.2)
Discontinued	1 (0.8)
Transitioned from gabapentin to pregabalin	1 (0.8)
Dose reduced	1 (0.8)
Indication documented for loop diuretic^a^	
Any	116 (96.7)
Lower-extremity swelling or edema	104 (86.7)
Congestive heart failure	16 (13.3)
Dyspnea	15 (12.5)
Hypertension	5 (4.2)
Swelling of other body part	6 (5.0)
Gabapentinoid-associated swelling	3 (2.5)
Other^c^	7 (5.8)
Selected diagnostic tests obtained for workup of edema	
Transthoracic echocardiography	22 (18.3)
Lower-extremity ultrasonography	5 (4.2)
Potential downstream event within 60 d^a^	
Any	28 (23.3)
Worsening kidney function^d^	9 (7.5)
Orthostasis	7 (5.8)
Electrolyte abnormality	6 (5.0)
Fall	5 (4.2)
Increased urinary frequency	4 (3.3)
Cramping	1 (0.8)
Drug allergy	1 (0.8)
Other downstream event^c^	4 (3.3)
Emergency department visit and/or hospitalization	6 (5.0)

^a^ Specific elements of the differential diagnosis, indication, and downstream events sum to a greater number than the overall category as multiple elements could have occurred for each patient (eg, a differential diagnosis with multiple considerations).

^b^ Nongabapentinoid adverse drug effects (n = 11) were attributed to calcium channel blockers (n = 7), carvedilol, mirtazapine, simeprevir, sofosbuvir, and testosterone.

^c^ Items in the other category for edema differential diagnosis, loop diuretic indication, and potential downstream events are detailed in eTable 2 in Supplement 1.

^d^ Worsening kidney function was defined as an increase in serum creatinine of (1) at least 0.3 mg/dL (22.9 µmol/L) or (2) an increase in serum creatinine to greater than 1.5 times baseline.

Clinicians almost universally documented the indication for LD (n = 116 [96.7%]), most commonly for lower-extremity edema (n = 104 [86.7%]), CHF (n = 16 [13.3%]), and/or dyspnea (n = 15 [12.5%]) (Table 2). Gabapentinoid-associated swelling was noted as the indication for LD in 3 patients (2.5%).

Actions taken toward the gabapentinoid prior to LD initiation occurred rarely (n = 8 [6.7%]) and involved dose increases (n = 5 [4.2%]), transition from gabapentin to pregabalin (n = 1 [0.8%]), discontinuation of the gabapentinoid (n = 1 [0.8%]), and dose reduction (n = 1 [0.8%]). Diagnostic workup for edema frequently included transthoracic echocardiography (n = 22 [18.3%]) and/or lower-extremity ultrasonography (n = 5 [4.2%]) (Table 2). Documentation of differential diagnoses, indications, and actions taken toward the gabapentinoid generally did not vary between patients with and without dementia (eTable 3 in Supplement 1).

### Potential Downstream Events

In the 60 days following LD initiation, 28 patients (23.3%) experienced 37 events potentially attributable to LD initiation (Table 2; Figure). The most common potential downstream events were worsening kidney function (n = 9 [7.5%]), orthostasis (n = 7 [5.8%]), electrolyte abnormalities (n = 6 [5.0%]), and falls (n = 5 [4.2%]). Of note, 6 patients (5.0%) presented to the emergency department and/or hospital for potential downstream events. Of the 37 events, 25 (67.6%) were judged to be possible and 10 (27.0%) to be probable in terms of attribution to LD use. Table 3 documents exemplar case vignettes.

**Figure.**
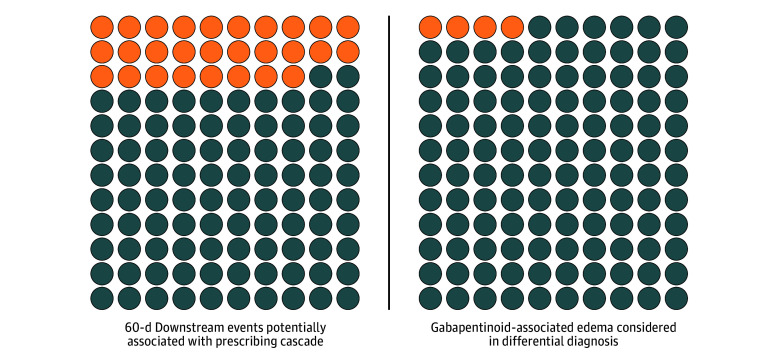
Icon Array of Key Study Findings (N = 120)

**Table 3.** Exemplar Case Vignettes

Category	Case vignette^a^
Care cascade including echocardiography and lower-extremity ultrasonography; probable diuretic-associated ADE	A man in his 60s was prescribed in month 1 gabapentin 900 mg nightly and 300 mg every morning for lower-extremity neuropathy attributed to chemotherapy for lung cancer. He developed lower-extremity edema, with normal echocardiography results obtained. In month 3, gabapentin was switched to pregabalin for ongoing neuropathy. In month 6, the patient was noted to have worsening bilateral lower-extremity edema, prompting lower-extremity ultrasonography, which was normal. He was started on furosemide 40 mg daily, and within 2 d, the patient developed lightheadedness and a sensation of being off balance, with improvement in symptoms after stopping furosemide. Rated probable diuretic-associated ADE because lightheadedness improved with cessation of furosemide and unlikely attributable to other processes.
Care cascade including echocardiography and lower-extremity ultrasonography; possible diuretic-associated ADE and ED visit	A man in his 60s with worsening back pain was evaluated by neurology in month 1 and prescribed pregabalin 75 mg twice a day. He was seen by primary care in month 2 and noted to have a recent onset of bilateral (right greater than left) lower-extremity edema. Lower-extremity ultrasonography was normal. The patient was prescribed furosemide 20 mg daily and underwent transthoracic echocardiography, which showed normal systolic function. In month 3, the patient presented to the ED with a noninjurious fall, and workup did not reveal fractures, but he required intravenous pain medication. Rated possible diuretic-associated ADE given that the fall was potentially attributable to multiple causes, including recent diuretic initiation, polypharmacy, Parkinson disease, and back pain.
Care cascade including echocardiography; possible diuretic-associated ADE	A man in his 70s was prescribed by primary care gabapentin 300 mg nightly for diabetic neuropathy in month 1. In month 2, he was evaluated by cardiology and found to have bilateral lower-extremity edema. The patient was prescribed furosemide 40 mg daily and, in parallel, underwent CHF workup. Echocardiography was notable for heart failure with preserved ejection fraction. In month 3, the patient developed hypokalemia, and furosemide was continued with potassium supplementation. Rated possible diuretic-associated ADE because furosemide was not withdrawn, so it was unclear whether the adverse event improved after drug discontinuation.
Probable diuretic-associated ADE	A man in his 80s developed back pain in month 1 and was prescribed gabapentin 300 mg twice a day by primary care. In month 2, he visited the primary care clinic for bilateral lower-extremity swelling of several days and was prescribed furosemide 20 mg daily. Two weeks later, the patient developed symptoms of itching, feeling “hot all over,” and a rash. He was switched from furosemide to chlorthalidone, with resolution of symptoms. Rated probable diuretic-associated ADE because symptoms improved with cessation of diuretic and other etiologies considered unlikely.
Recognition of potential prescribing cascade	A man in his 80s with dementia was prescribed by a geriatrician (by telephone) gabapentin 300 mg 3 times daily for diabetic peripheral neuropathy in month 1. In month 2, he was evaluated in the geriatrics clinic and found to have bilateral lower-extremity and hand swelling. Clinician suspected that the swelling could be associated with gabapentin, and recommended tapering off gabapentin while simultaneously initiating furosemide 20 mg daily. The patient was evaluated again in the clinic in month 5, with swelling improved to minimal ankle edema. He was no longer taking gabapentin and continued treatment with furosemide. No downstream events were noted.

Abbreviations: ADE, adverse drug event; CHF, congestive heart failure; ED, emergency department.

^a^ In each vignette, the starting point (ie, prescription of gabapentinoid) was standardized to month 1 to make it easier to follow timelines across multiple cases.

Potential downstream events generally did not vary between patients with and without dementia, although there was a higher percentage of patients with dementia who experienced an emergency department visit and/or hospitalization related to a potential adverse drug event (4 patients [11.8%] vs 2 patients [2.3%], respectively) (eTable 3 in Supplement 1). In exploratory analyses, we did not find evidence that age, comorbidity burden, or dementia diagnosis were associated with the presence of documented differential diagnoses or potential downstream events following LD initiation (eTable 4 in Supplement 1).

## Discussion

This cohort study found that clinicians almost never explicitly considered gabapentinoid-related adverse drug effects in their treatment of edema in older, mostly male veterans who received an LD after gabapentinoid initiation and potentially experienced the gabapentinoid-LD prescribing cascade. Clinicians commonly weighed alternative processes, such as CHF, venous stasis, and/or nongabapentinoid-related adverse drug effects, highly in their clinical reasoning. Potential downstream harms of the gabapentinoid-LD prescribing cascade were common, occurring in nearly one-quarter of patients and, in some cases, precipitating emergency department visits and/or hospitalizations. These findings underscore the potential for prescribing cascades, such as the gabapentinoid-LD cascade, to propagate unintentionally and to potentiate important drug-related harms among older adults. Importantly, we were unable to discover how often clinicians correctly recognized edema as a gabapentinoid adverse effect and stopped the drug without starting an LD since such individuals were not included in our cohort.

By leveraging a national sample, our findings extend those of a few smaller studies using medical record review and/or qualitative methods to unpack the clinical contexts and decision-making processes associated with prescribing cascades.^31,32^ Farrell and colleagues^9,10^ conducted semistructured interviews with older adults possibly affected by prescribing cascades and their caregivers and clinicians. Participants revealed that prescribing cascades were difficult to identify in clinical practice and that their development was associated with varying levels of awareness about medications and medication-related adverse effects. Moreover, investigators emphasized the challenges of confirming prescribing cascades due to a lack of documented information regarding medication indications, chronologies of prescribing decisions, and data regarding prior attempts to deprescribe or reduce dosing of the initial medication in the cascade.

With these prior studies in mind, we assembled our cohort to encompass individuals in whom the gabapentinoid-LD cascade was plausible, meaning that it would be reasonable based on the timing of the initiation of medications and the occurrence of edema for a clinician to consider gabapentinoid-associated edema in their differential diagnosis and subsequent management. In line with these prior studies, we could not confirm that prescribing cascades definitively occurred in these patients. Nevertheless, of 120 patients, gabapentinoid-associated edema was considered in only a handful. Moreover, actions involving dose reductions and/or discontinuation of gabapentinoids, a key component of confirming the existence of a prescribing cascade,^10^ were exceedingly rare. Clinicians more frequently uptitrated gabapentinoid dosing in the setting of edema rather than decreased the dosing, suggesting that this potential drug-related adverse effect was overlooked in many cases. While in some patients edema may have been attributable to processes other than a drug-related adverse effect (eg, truly new-onset CHF or venous stasis), it was also plausible, and in most cases entirely unexplored, that the gabapentinoid-associated adverse effect was a primary or contributing factor. In contrast, a greater number of clinicians raised the possibility of calcium channel blocker–associated edema, which may reflect that there was greater attention paid to the association between calcium channel blocker use (compared with gabapentinoid) and edema and the promising notion that this evidence base has had longer to influence clinical practice.^4,33,34^ Of note, we observed the gabapentinoid-LD prescribing cascade at relatively modest gabapentinoid doses. Prior work found that while edema may develop at any gabapentinoid dose, it is more common at higher doses.^14,20^ Therefore, clinicians in our study may have had a lower level of suspicion for gabapentinoid-associated edema.

Rather than excluding a medication-associated cause for new edema (ie, through deprescribing or dose reduction of gabapentinoid), clinicians far more frequently considered and acted on other potential etiologies for lower-extremity edema, often leading to diagnostic studies such as echocardiography and lower-extremity ultrasonography. Beyond simply being unaware of the potential for gabapentinoids to cause edema and/or overlooking a possible drug-associated etiology, there are several potential reasons for this finding. First, clinicians may prioritize do-not-miss diagnoses such as CHF, especially in older patients with substantial comorbidity burden and cardiovascular risk.^35^ Second, clinicians and patients may have a bias toward action-oriented steps (ie, ordering tests, starting medications) rather than systematically reviewing and addressing potential medication-associated harms, despite that testing may, in turn, precipitate incidental findings, care cascades, and potentially unnecessary treatment.^36^ Third, given serious time constraints, it may be more expedient to pursue additional testing rather than undertake time-consuming, in-depth medication reconciliation and subsequent optimization.^10^ Finally, our finding that nearly two-thirds of patients had discordant prescribers of gabapentinoid and LD lends credence to the notion that suboptimal awareness of prescribing decisions across fragmented teams may be an important driver of prescribing cascades.^37^

Our study found that potential downstream harms, such as electrolyte abnormalities and falls, associated with the gabapentinoid-LD prescribing cascade occurred in nearly one-quarter of patients. This finding reinforces prior research documenting adverse events associated with various prescribing cascades, ranging from decreased quality of life to serious adverse events such as emergency department visits and hospitalizations.^38,39^ Our study focused on individuals who initiated both gabapentinoid and LD, and we did not have a comparator group in which to measure adverse effects (ie, a group that had not been exposed to a potential prescribing cascade). To disentangle drug effects from other processes, we conducted a causality assessment, finding that more than one-quarter of these events were probable in terms of drug-related effects. Given the ubiquity of gabapentinoid use among older adults and how infrequently gabapentinoid-associated processes were considered, these results underscore the importance of elevating drug-related harms in clinical reasoning and the promise of novel tools to mitigate prescribing cascades in clinical practice.^1,2,11,40^

The secondary aim of our study was to investigate the association of a dementia diagnosis with the decision-making processes leading to prescribing cascades and downstream outcomes. People with dementia and/or their caregivers may face unique challenges in recognizing and reporting adverse effects from medications due to cognitive impairment, and their clinicians may be less likely to diagnose drug-related harms as a result.^41,42^ Nevertheless, we generally did not find significant differences between patients with and without dementia in terms of key decision-making steps along the cascade and in terms of downstream outcomes. While we enriched our medical record review sample for patients with dementia, we still had a small sample size for comparison, limiting power to detect small differences. Similarly, our exploratory analysis did not find age, comorbidity burden, or a diagnosis of dementia to be associated with documented differential diagnoses or downstream events.

### Limitations

This study had several limitations. First, data regarding decision-making and clinical reasoning emerged from review of clinical documentation; therefore, we were unable to assess other considerations that may have been in the minds of clinicians involved in each case but were not documented. Nevertheless, in most cases, we noted some documentation regarding the differential diagnosis of edema and near-universal documentation of indications for LD use, providing important context for rare references to potential gabapentinoid-associated edema. Furthermore, medical record review allowed us to discern critical contextual information and certain outcomes, such as falls, that may not be fully coded in administrative data.^43^ Second, our analysis focused on individuals who potentially experienced the prescribing cascade; thus, we were unable to comment on how often clinicians did not prescribe LD for potential gabapentinoid-induced edema (ie, prevented the prescribing cascade). However, a large share of gabapentinoid-associated edema may be unrecognized and not addressed through reduction or cessation of gabapentinoids as clinicians often overlook adverse drug events in older adults.^44,45^ Nevertheless, the estimated rates of documentation practices and downstream consequences may not reflect the full spectrum of prescribing cascade responses in clinical practice. Finally, our study drew from the VA health system, the largest integrated health system in the US, which serves a predominantly male population; thus, our findings may not be transferable to female patients or other health care settings. Nevertheless, use of VA data allowed for random selection from a national sample and powerful linkages of administrative and clinical data.

## Conclusions

In this cohort study of older veterans who potentially experienced the gabapentinoid-LD prescribing cascade, structured analysis of clinical documentation revealed that clinicians almost never explicitly considered gabapentinoid adverse drug effects in their management of edema and that potential downstream harms were relatively common. Our findings underscore the importance of further elucidating the decision-making processes underlying prescribing cascades beyond documentation (eg, via qualitative interviews with prescribers and patients) and developing and testing interventions to recognize, prevent, and mitigate prescribing cascades in clinical practice.

## Notes

Supplement 1.
eMethods.

eReferences.
**eAppendix.** Sample Standardized Abstraction Form **eFigure.** Cohort Flow Diagram **eTable 1.** Sample Characteristics by Dementia Status **eTable 2.** Items in Other Category for Edema Differential Diagnosis, Loop Diuretic Indications, and Potential Downstream Events **eTable 3.** Key Decision-Making Steps and Potential Downstream Outcomes of Gabapentinoid–Loop Diuretic Prescribing Cascade by Dementia Status **eTable 4.** Assessment of Associations Between Selected Patient Characteristics and Documented Differential Diagnoses of Potential Downstream Events Following Loop Diuretic Initiation

Supplement 2.
Data Sharing Statement
