# Supplementary material for: Decision-Making and Downstream Outcomes of the Gabapentinoid-Diuretic Prescribing Cascade
Source: JAMA Netw Open. 2025 Dec 2;8(12):e2545274. doi: 10.1001/jamanetworkopen.2025.45274 (PMC12673415; doi:10.1001/jamanetworkopen.2025.45274)
Supplement: Supplement 1. — eMethods. eReferences. eAppendix. Sample Standardized Abstraction Form eFigure. Cohort Flow Diagram eTable 1. Sample Characteristics by Dementia Status eTable 2. Items in Other Category for Edema Differential Diagnosis, Loop Diuretic Indications, and Potential Downstream Events eTable 3. Key Decision-Making Steps and Potential Downstream Outcomes of Gabapentinoid–Loop Diuretic Prescribing Cascade by Dementia Status eTable 4. Assessment of Associations Between Selected Patient Characteristics and Documented Differential Diagnoses of Potential Downstream Events Following Loop Diuretic Initiation [file jamanetwopen-e2545274-s001.pdf]

Supplemental Online Content

Growdon ME, Tjota N, Campbell R, et al. Decision-making and downstream outcomes of the gabapentinoid-diuretic prescribing cascade. *JAMA Netw Open*. 2025;8(11):e2545274. doi:10.1001/jamanetworkopen.2025.45274

eMethods

eReferences

eAppendix. Sample Standardized Abstraction Form

eFigure. Cohort Flow Diagram

eTable 1. Sample Characteristics by Dementia Status

eTable 2. Items in Other Category for Edema Differential Diagnosis, Loop Diuretic Indications, and Potential Downstream Events

eTable 3. Key Decision-Making Steps and Potential Downstream Outcomes of Gabapentinoid–Loop Diuretic Prescribing Cascade by Dementia Status

eTable 4. Assessment of Associations Between Selected Patient Characteristics and Documented Differential Diagnoses of Potential Downstream Events Following Loop Diuretic Initiation

This supplemental material has been provided by the authors to give readers additional information about their work.

© 2025 Growdon ME et al. *JAMA Network Open*.

eMethods

Additional Chart Review Methods

Abstractors viewed medical records using the Veterans Affairs (VA) Joint Longitudinal Viewer (JLV), an integrated, read-only interface with national health data from VA, Department of Defense, VA community partners, and scanned documents from many non-VA healthcare encounters.[1] JLV includes a broad array of patient-level health information, including outpatient and inpatient medications (including fields available in the VA in which medication indications can be manually documented), orders, laboratory values, and radiology records. Abstractors reviewed all clinical documentation in the applicable time frame for each patient. For information related to differential diagnosis and indication for loop diuretic prescription, review focused on documentation generated by the prescribing clinician (including but not limited to notes, telephone encounters, correspondence, and secure messaging with the patient). We reviewed notes from other providers and clinical data as relevant for other data elements (e.g., diagnostic testing, downstream events potentially related to loop diuretic). A sample of the standardized VA Research Electronic Data Capture (REDCap) abstraction form is included in the eAppendix.

For identification of worsening kidney function, we relied on the 2012 Kidney Disease: Improving Global Outcomes (KDIGO) definition, which defines acute kidney injury as 1) an increase in serum creatinine greater than or equal to 0.3 mg/dl and/or 2) an increase in serum creatinine to greater than 1.5 times baseline.[2] Given that in the outpatient setting serum creatinine is generally not checked frequently, and in keeping with prior studies of acute kidney injury in the predominantly outpatient setting, we defined the baseline creatinine value based on the lowest serum creatinine value in the twelve months prior to the value associated with the potential downstream event.[3] This definition generally aligns with a consensus definition of "worsening kidney function" used in other studies.[4] For falls, we counted any mention of a fall, which we rated as injurious or non-injurious, regardless of whether a patient had experienced prior falls. For electrolyte abnormalities, we considered values outside of the reference range to be abnormal (e.g., for sodium, potassium, and magnesium).

Key Variables

Race/ethnicity categories were based on the Research Triangle Institute definitions found in Medicare claims. We calculated the Charlson comorbidity index from VA and Medicare claims during the 2 years before initiation of gabapentinoid.[5] We used a 3-year look-back period in claims for dementia diagnosis, in line with prior studies.[6] We defined baseline chronic medication use as those with fills of greater than or equal to 14 pills in the 6 months prior to gabapentinoid initiation to exclude very short-term or highly intermittent use.[7]

eReferences

1. Joint Longitudinal Viewer (JLV) and Research Part 1: Getting Started. March 18, 2025. Accessed April 16, 2025. https://www.hsrd.research.va.gov/for_researchers/cyber_seminars/archives/video_archive.cfm?SessionID=5182

2. Kidney Disease: Improving Global Outcomes (KDIGO) Acute Kidney Injury Work Group. KDIGO Clinical Practice Guideline for Acute Kidney Injury. *Kidney Inter, Suppl*. 2012;2:1-138. doi:10.1038/kisup.2012.1

3. Hobbs H, Bassett P, Wheeler T, et al. Do acute elevations of serum creatinine in primary care engender an increased mortality risk? *BMC Nephrology*. 2014;15(1):206. doi:10.1186/1471-2369-15-206

4. Lala A, Coca S, Feinman J, et al. Standardized Definitions of Changes in Kidney Function in Trials of Heart Failure. *JACC*. 2025;85(7):766-781. doi:10.1016/j.jacc.2024.11.041

5. Deyo RA, Cherkin DC, Ciol MA. Adapting a clinical comorbidity index for use with ICD-9-CM administrative databases. *J Clin Epidemiol*. 1992;45(6):613-619. doi:10.1016/0895-4356(92)90133-8

6. Maust DT, Strominger J, Bynum JPW, et al. Prevalence of Psychotropic and Opioid Prescription Fills Among Community-Dwelling Older Adults With Dementia in the US. *JAMA*. 2020;324(7):706-708. doi:10.1001/jama.2020.8519

7. Anderson TS, Jing B, Wray CM, et al. Comparison of pharmacy database methods for determining prevalent chronic medication use. *Med Care*. 2019;57(10):836-842. doi:10.1097/MLR.0000000000001188. PMCID: PMC6742560.

© 2025 Growdon ME et al. *JAMA Network Open*.

eAppendix. Sample Standardized Abstraction Form

Record ID:

Reviewer's initials:

Linked ID:

PART ONE OF CHART REVIEW: FOCUSED ON TIME LEADING UP TO LOOP DIURETIC FILL (Primary chart review will be targeted to 30 days leading up and including index date)

Date of loop diuretic fill: (M-D-Y)

In the 30 days leading up to and including the date of loop diuretic fill, was there a clear indication that the chart should be EXCLUDED for further review due to any of the following (select all that apply)?

[]Clear indication that gabapentinoid was stopped by patient >=30 days before loop diuretic was prescribed (exclude chart)
[]Chart lacks any reference to lower extremity edema/swelling(exclude chart)
[]Chart indicates that patient was already taking loop diuretic (eg, from non VA pharmacy/provider or recently started in an outside system/hospital) and therefore this is not new prescribing of loop diuretic (exclude chart)
[]None of the other options apply (continue with chart review)

Was a reason/indication given for prescribing the loop diuretic? Y/N

Reason(s) given for prescribing of loop diuretic (select all that apply):
[]Lower extremity swelling/edema
[]Swelling of any other body part
[]Adverse drug event related to gabapentinoid
[]Dyspnea / shortness of breath
[]Congestive heart failure (CHF)
[]Hypertension
[]Other

Reason for Other (for reason for prescribing diuretic):

Was any differential diagnosis for edema documented? Y/N

Documented differential diagnosis for edema (check all that apply):

[]Adverse drug event related to gabapentinoid
[]Congestive heart failure/CHF
[]Venous stasis
[]Non-gabapentinoid-related adverse drug effect
[]Deep venous thrombosis/DVT
[]Other

Reason for "Other" (for differential diagnosis of edema):

Was any action taken towards the gabapentinoid? Y/N

Action towards gabapentinoid:
[]Downtitrate dose
[]Discontinue
[]Transition from gabapentin→pregabalin
[]Increase dose

PART TWO OF CHART REVIEW: FOCUSED ON TIME LEADING UP TO GABAPENTINOID FILL

Date of gabapentinoid fill: (M-D-Y)

© 2025 Growdon ME et al. *JAMA Network Open*.

Was there a reference in the chart to the patient having a history/diagnosis of congestive heart failure in the 30 days leading up to and including the date of gabapentinoid fill? (e.g., in problem list, plan, one-liner, etc.) Y/N

Diagnostic tests obtained for work-up of edema (select all that apply):
[]Echocardiogram
[]Lower extremity ultrasound

In the 60 DAYS after the loop diuretic was filled, did a possible downstream event (fall, dizziness, electrolyte abnormality, urinary urgency/symptoms, ED or hospitalization related to any of those) occur? Y/N

PART THREE OF CHART REVIEW: FOCUSED ON 60 DAYS AFTER LOOP DIURETIC FILL

Possible downstream effect(s) (select all that are applicable):

[]Orthostasis/dizziness
[]Electrolyte abnormality (hyponatremia, hypokalemia, hypomagnesemia)
[]Worsening kidney function
[]Fall (specify injurious vs non-injurious)
[]Cramping
[]Allergic reaction (please specify symptoms below)
[]Increased urinary frequency
[]Other

Reason for Other (Downstream effect):

For any potential downstream harms identified, please provide date and brief description of potential downstream harm (e.g, 1/1/2018 - fall noted in PCP note). If there were multiple potential downstream events, please provide as much detail as possible for each.

For the above identified downstream outcome(s), please assess its/their WHO causality category:

Unlikely:
• Event or laboratory test abnormality, with a time to drug intake that makes a relationship improbable (but not impossible)
• Disease or other drugs provide plausible explanations

Possible:
• Event or laboratory test abnormality, with reasonable time relationship to drug intake
• Could also be explained by disease or other drugs
• Information on drug withdrawal may be lacking or unclear

Probable/Likely:
• Event or laboratory test abnormality, with reasonable time relationship to drug intake
• Unlikely to be attributed to disease or other drugs
• Response to withdrawal clinically reasonable
• Rechallenge not required

Certain:
• Event or laboratory test abnormality, with plausible time relationship to drug intake
• Cannot be explained by disease or other drugs
• Response to withdrawal plausible (pharmacologically, pathologically)
• Event definitive pharmacologically or phenomenologically (i.e. an objective and specific medical disorder or a recognised pharmacological phenomenon)
• Rechallenge satisfactory, if necessary

Unclassifiable:
• Report suggesting an adverse reaction
• Cannot be judged because information is insufficient or contradictory
• Data cannot be supplemented or verified

In the 60 days following dispensing of the loop diuretic, was there an ED visit or hospitalization related to any of the selected downstream events? Y/N

© 2025 Growdon ME et al. *JAMA Network Open*.

Details about ED visit(s) or hospitalization(s) (date, reason, copied notes):

Any additional notes (eg details that were unclear or may need secondary review, etc.):

Date Completed:

© 2025 Growdon ME et al. *JAMA Network Open*.

eFigure. Cohort Flow Diagram

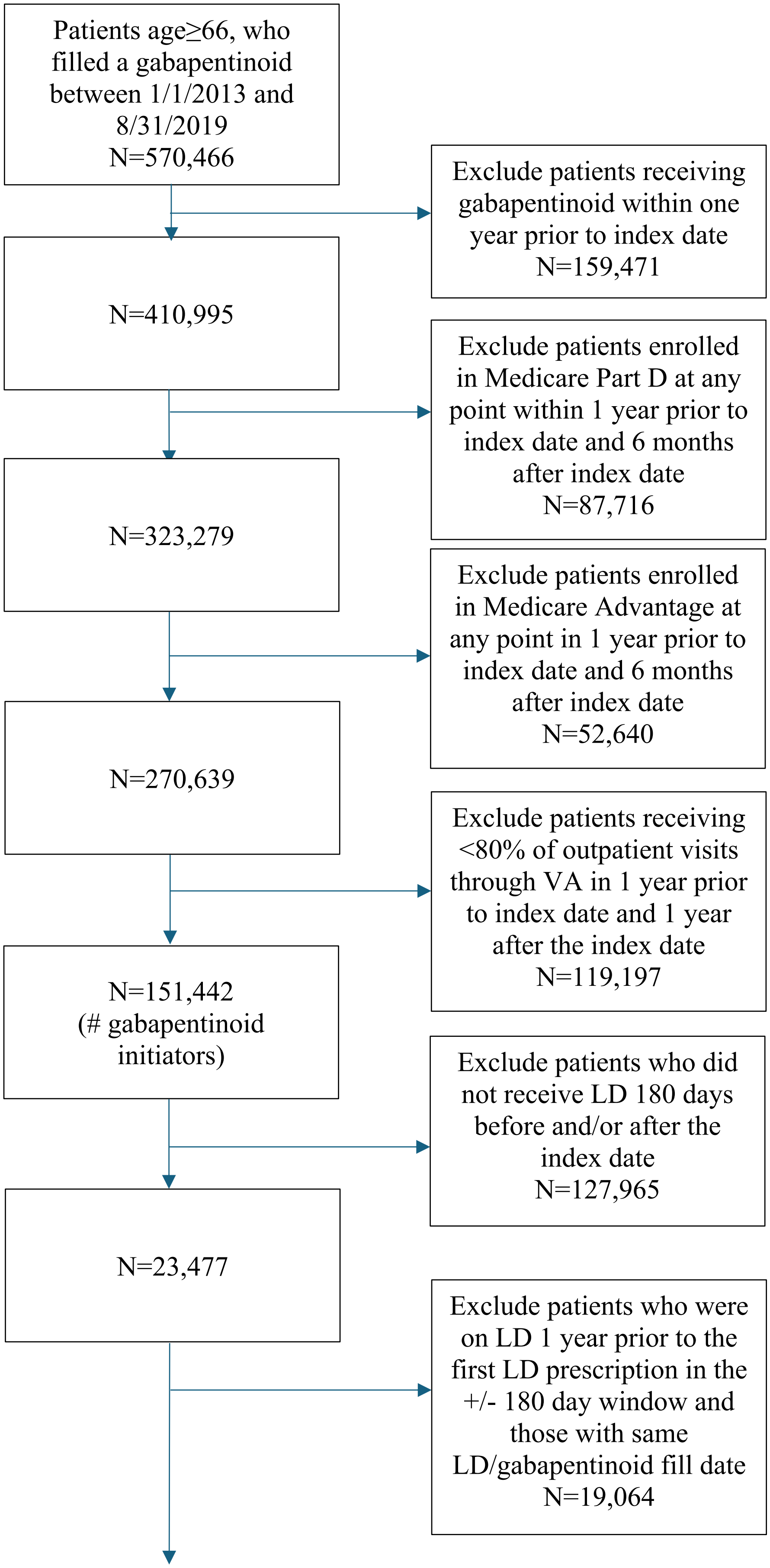

© 2025 Growdon ME et al. *JAMA Network Open*.

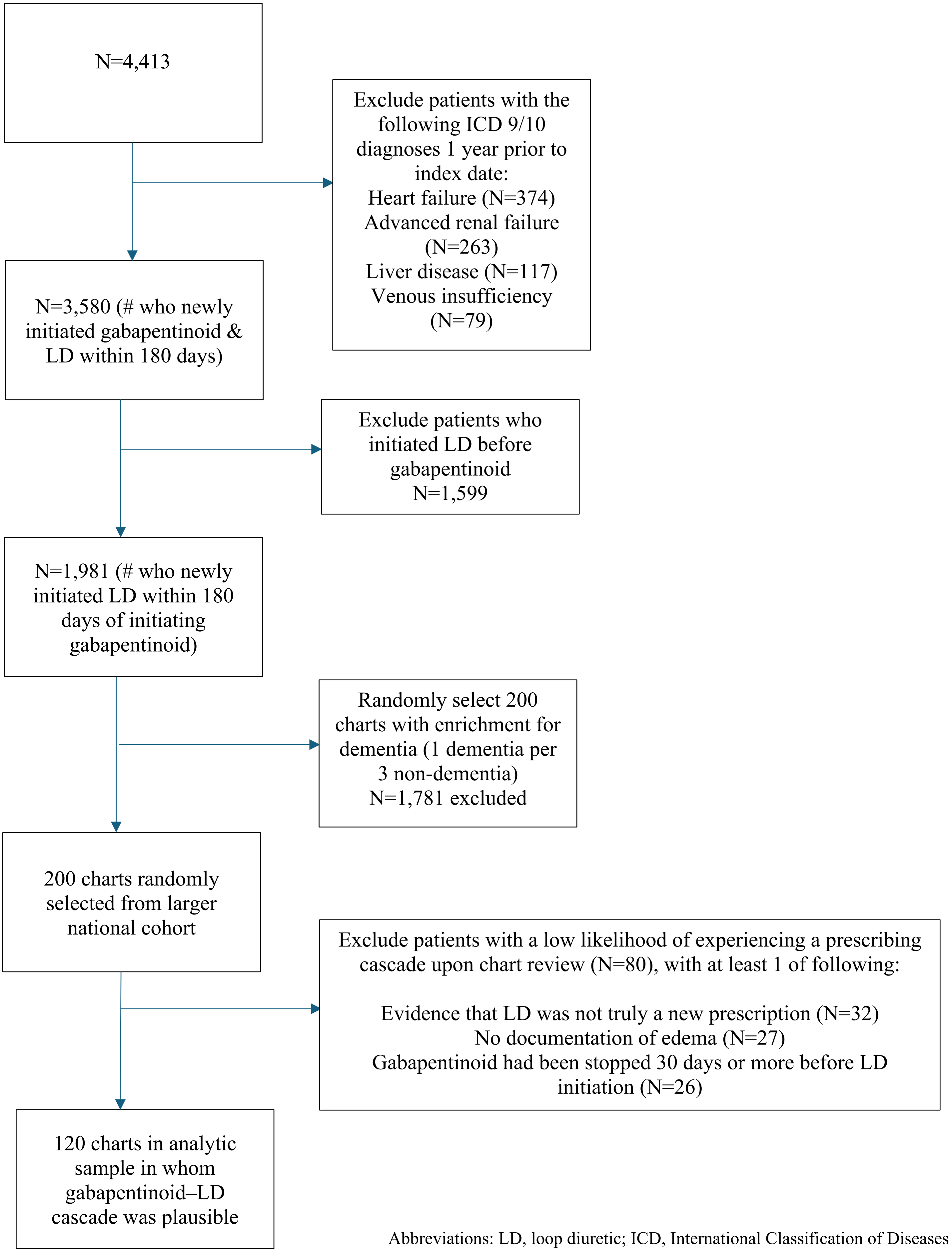

© 2025 Growdon ME et al. *JAMA Network Open*.

eTable 1. Sample Characteristics by Dementia Status

Characteristic[a]	Patients, No. (%) N=120	Patients without dementia, No. (%) N=86 (72%)	Patients with dementia, No. (%) N=34 (28%)	p-value
**Age, years (mean, SD)**	73.9 (7.1)	72.3 (6.3)	77.9 (7.6)	<0.001
65-74	74 (62)	62 (72)	12 (35)	
74-84	33 (28)	19 (22)	14 (41)	
85+	13 (11)	5 (6)	8 (24)	
**Sex: Female**	4 (3)	3 (4)	1 (3)	0.88
**Race/Ethnicity**				
Black, Non-Hispanic	13 (11)	5 (6)	8 (24)	0.01
Hispanic	6 (5)	6 (7)	0 (0)	
White, Non-Hispanic	98 (82)	72 (84)	26 (77)	
Other[b]	3 (3)	3 (4)	0 (0)	
**Charlson Comorbidity Index, median (IQR)**	3 (1,6)	3 (1.5)	4.5 (3,7)	0.008
**Baseline medication count ≥5**	106 (88)	78 (91)	28 (82)	0.19
**Hospitalization in last year**	23 (19)	14 (16)	9 (27)	0.20
**Clinical encounters in past year, median (IQR), No.**	25 (14,44)	24 (14, 42)	32 (18, 53)	0.07
**Types of specialty clinics in last year, median (IQR), No.**	4 (3,6)	4 (3,6)	5 (3,7)	0.11
**Different clinicians prescribed gabapentinoid and diuretic**	72 (60.0)	52 (60)	20 (59)	0.87

Abbreviations: SD, standard deviation; IQR, interquartile range.

[a]Sample characteristics were derived from VA and Medicare administrative data.

[b]Includes American Indian or Alaska Native, Asian or Pacific Islander, other, or unknown.

© 2025 Growdon ME et al. *JAMA Network Open*.

eTable 2. Items in Other Category for Edema Differential Diagnosis, Loop Diuretic Indications, and Potential Downstream Events

Differential diagnosis for edema[a]
Low albumin
Familial amyloidosis
Stopping hydrochlorothiazide
Chronic obstructive pulmonary disease
Arterial insufficiency
Sleeping in chair
Cardiac issues
Chronic kidney disease
Dependent edema
Suboptimally controlled hypertension
Lymphedema
Gout
Pneumonia with pleural effusion
Hepatic disease
Cellulitis
Severe anemia
Obstructive sleep apnea
Deconditioning
**Indication for loop diuretic**
Pleural effusions
Dependent edema from sleeping on one side
Pneumonia with pleural effusion
Obesity
Fatigue
Wound dehiscence
Hypercalcemia
**Potential 60-day downstream events related to prescribing cascade**
Loose stools
Worsening neuropathy
Hypotension
Failure to thrive

[a]We generally classified items into the "Other" category if they were not included in the explicit criteria of the chart abstraction guide, which was iteratively developed during the initial phase of the chart review.

© 2025 Growdon ME et al. *JAMA Network Open*.

eTable 3. Key Decision-Making Steps and Potential Downstream Outcomes of Gabapentinoid–Loop Diuretic Prescribing Cascade by Dementia Status

Characteristic	Patients, No. (%) N=120	Patients without dementia, No. (%) N=86 (72%)	Patients with dementia, No. (%) N=34 (28%)	p-value[a]
**Differential diagnosis documented for edema**	73 (61)	53 (62)	20 (59)	0.78
**Differential diagnosis includes**				
Congestive heart failure	47 (39)	32 (37)	15 (44)	0.49
Venous stasis	16 (13)	12 (14)	4 (12)	0.75
Non-gabapentinoid drug side effect	11 (9)	8 (9)	3 (9)	0.94
Deep venous thrombosis	7 (6)	6 (7)	1 (3)	0.40
Gabapentinoid-related side effect	4 (3)	2 (2)	2 (6)	0.33
Other	18 (15)	16 (19)	2 (6)	0.08
**Any action taken towards gabapentinoid prior to loop diuretic initiation**	8 (7)	5 (6)	3 (9)	0.55
**Gabapentinoid action**				
No action	112 (93)	80 (93)	32 (94)	0.48
Dose increased	5 (4)	4 (5)	1 (3)	
Discontinued	1 (1)	1 (1)	0 (0)	
Transitioned from gabapentin to pregabalin	1 (1)	1 (1)	0 (0)	
Dose reduction	1 (1)	0 (0)	1 (1)	
**Indication documented for loop diuretic**	116 (97)	85 (99)	31 (91)	0.04
Lower extremity swelling/edema	104 (87)	77 (90)	27 (79)	0.14
Congestive heart failure	16 (13)	11 (13)	5 (15)	0.78
Dyspnea	15 (13)	12 (14)	3 (9)	0.44
Hypertension	5 (4)	4 (5)	1 (3)	0.67
Swelling of other body part	6 (5)	2 (2)	4 (12)	0.03
Gabapentinoid-related swelling	3 (3)	1 (1)	2 (6)	0.14
Other	7 (6)	5 (6)	2 (6)	0.99
**Selected diagnostic tests obtained for workup of edema**				
Transthoracic echocardiogram	22 (18)	17 (20)	5 (15)	0.52
Lower extremity ultrasound	5 (4)	4 (5)	1 (3)	0.67
**Any potential downstream event within 60 days**	28 (23)	20 (23)	8 (24)	0.98
Worsening kidney function	9 (8)	7 (8)	2 (6)	0.67
Orthostasis	7 (6)	5 (6)	2 (6)	0.99
Electrolyte abnormality	6 (5)	6 (7)	0 (0)	0.11
Fall	5 (4)	3 (4)	2 (6)	0.55
Cramping	1 (1)	1 (1)	0 (0)	0.53

© 2025 Growdon ME et al. *JAMA Network Open*.

Characteristic	Patients, No. (%) N=120	Patients without dementia, No. (%) N=86 (72%)	Patients with dementia, No. (%) N=34 (28%)	p-value[a]
Drug allergy	1 (1)	1 (1)	0 (0)	0.53
Increased urinary frequency	4 (3)	2 (2)	2 (6)	0.33
Other downstream event	4 (3)	2 (2)	2 (6)	0.33
Emergency department visit and/or hospitalization related to above	6 (5)	2 (2)	4 (12)	0.03

[a]P values should be interpreted judiciously given multiple hypothesis testing.

© 2025 Growdon ME et al. *JAMA Network Open*.

eTable 4. Assessment of Associations Between Selected Patient Characteristics and Documented Differential Diagnoses or Potential Downstream Events Following Loop Diuretic Initiation

	Odds ratio for outcome of presence of differential diagnosis		Odds ratio for outcome of presence of at least one downstream event	
**Variable**	**Univariable**	**Multivariable[a]**	**Univariable**	**Multivariable[a]**
**Age, years [ref: 66-74]**				
75-84	0.94 (0.40-2.2)	0.96 (0.40-2.3)	1.1 (0.41-2.8)	1.0 (0.37-2.8)
85+	0.71 (0.22-2.3)	0.73 (0.21-2.6)	1.0 (0.25-4.1)	0.97 (0.22-4.2)
**Dementia**	0.89 (0.40-2.0)	0.96 (0.40-2.3)	1.0 (0.40-2.6)	0.94 (0.34-2.6)
**Charlson Comorbidity Index ≥ median (3)**	0.91 (0.43-1.9)	0.94 (0.43-2.1)	1.4 (0.55-3.3)	1.4 (0.54-3.5)

[a]Multivariable models were adjusted for 3 variables chosen *a priori*: age, dementia diagnosis, and Charlson Comorbidity Index (dichotomized at the median value in the sample).

© 2025 Growdon ME et al. *JAMA Network Open*.
