# Supplementary material for: Decision-Making and Downstream Outcomes of the Gabapentinoid-Diuretic Prescribing Cascade
Source: JAMA Netw Open. 2025 Dec 2;8(12):e2545274. doi: 10.1001/jamanetworkopen.2025.45274 (PMC12673415; doi:10.1001/jamanetworkopen.2025.45274)
Supplement: Supplement 2. — Data Sharing Statement [file jamanetwopen-e2545274-s002.pdf]

Data Sharing Statement

Data
Data available: No

Additional Information
Explanation for why data not available: No additional data are available for sharing owing to a data use agreement with the US Department of Veterans Affairs. The statistical code used in programming and/or analysis can be made freely available to others.
